# Altered Insulin Receptor Signalling and β-Cell Cycle Dynamics in Type 2 Diabetes Mellitus

**DOI:** 10.1371/journal.pone.0028050

**Published:** 2011-11-30

**Authors:** Franco Folli, Terumasa Okada, Carla Perego, Jenny Gunton, Chong Wee Liew, Masaru Akiyama, Anna D'Amico, Stefano La Rosa, Claudia Placidi, Roberto Lupi, Piero Marchetti, Giorgio Sesti, Marc Hellerstein, Lucia Perego, Rohit N. Kulkarni

**Affiliations:** 1 Division of Diabetes, Department of Medicine, University of Texas Health Science Center, San Antonio, Texas, United States of America; 2 Research Division, Joslin Diabetes Center and Department of Medicine, Harvard Medical School, Boston, Massachusetts, United States of America; 3 Department of Molecular Science Applied to Biosystems, Università degli Studi di Milano, Milan, Italy; 4 Garvan Institute of Medical Research, Sydney, Australia; 5 Department of Pathology, Ospedale di Circolo and Department of Human Morphology, University of Insubria, Varese, Italy; 6 Division of Endocrinology, University of Pisa, Pisa, Italy; 7 Department of Clinical and Experimental Medicine, University Magna Graecia of Catanzaro, Catanzaro, Italy; 8 Department of Nutritional Sciences and Toxicology, University of California, Berkeley, California, United States of America; Mayo Clinic College of Medicine, United States of America

## Abstract

Insulin resistance, reduced β-cell mass, and hyperglucagonemia are consistent features in type 2 diabetes mellitus (T2DM). We used pancreas and islets from humans with T2DM to examine the regulation of insulin signaling and cell-cycle control of islet cells. We observed reduced β-cell mass and increased α-cell mass in the Type 2 diabetic pancreas. Confocal microscopy, real-time PCR and western blotting analyses revealed increased expression of PCNA and down-regulation of p27-Kip1 and altered expression of insulin receptors, insulin receptor substrate-2 and phosphorylated BAD. To investigate the mechanisms underlying these findings, we examined a mouse model of insulin resistance in β-cells – which also exhibits reduced β-cell mass, the β-cell-specific insulin receptor knockout (βIRKO). Freshly isolated islets and β-cell lines derived from βIRKO mice exhibited poor cell-cycle progression, nuclear restriction of FoxO1 and reduced expression of cell-cycle proteins favoring growth arrest. Re-expression of insulin receptors in βIRKO β-cells reversed the defects and promoted cell cycle progression and proliferation implying a role for insulin-signaling in β-cell growth. These data provide evidence that human β- and α-cells can enter the cell-cycle, but proliferation of β-cells in T2DM fails due to G1-to-S phase arrest secondary to defective insulin signaling. Activation of insulin signaling, FoxO1 and proteins in β-cell-cycle progression are attractive therapeutic targets to enhance β-cell regeneration in the treatment of T2DM.

## Introduction

Type 2 diabetes mellitus results from a combination of insulin resistance, defects in insulin secretion and hyperglucagonemia. It has been recognized for several decades that overt type 2 diabetes is delayed for a considerable period by the ability of pancreatic islets to compensate for the ambient insulin resistance, glucose toxicity and amyloid deposition [1], [2], [3], [4], [5], [6], [7], [8], [9], [10]. However, the pathways that underlie these failed compensatory mechanisms in humans are not fully defined. Longitudinal studies in rodent models of diabetes clearly indicate that islets can compensate for the insulin resistance by replication and neogenesis [11], [12], [13]. An increase in β-cell volume in insulin-resistant obese non-diabetic humans suggests that an islet compensation likely occurs in humans as an initial adaptive response to insulin resistance, to delay the onset of hyperglycemia in type 2 diabetes [14], [15]. However, examination of dynamic changes in β-cell mass in humans is limited by the techniques available to gain experimental access to islets *in vivo*. Thus, most studies to date have focused on studying post-mortem pancreas sections [12], [14], [15], [16], [17] and in some cases using islets isolated from patients with type 2 diabetes [18], [19], [20], [21].

β-cell mass is regulated by a balance between generation of new β-cells (by replication and neogenesis) and β-cell death (apoptosis) [21], [22], [23], [24]. Most studies to date suggest that a decrease in β-cell mass in established cases of human and non-human primates affected by type 2 diabetes is due to enhanced apoptosis [6], [15], [16]. Interestingly, studies in rodents indicate that adult β-cell mass is maintained by replication of existing β-cells involving cyclin D1 and D2 or both [22], [25], [26], [27]. A role for β-cell replication is also evident in the compensatory response to insulin resistance that has been linked to the homeodomain transcription factor PDX-1 and the forkhead transcription factor [28], [29]. Other studies in rodents and in human islet precursor cells suggest that a reversible process of de-differentiation and differentiation contributes to β-cell expansion, and new β-cells may form by transdifferentiation [30], [31], [32]. While studies in both rodents and humans indicate a role for insulin signaling proteins in the modulation of islet secretion and function, and growth factor inhibitors have been suggested to play a role during differentiation of embryonic stem cells, evidence for a potential link between altered growth factor signaling and β-cell regeneration in the context of type 2 diabetes is not fully understood [9], [13], [19], [33], [34].

In this study we have examined pancreas and islets from patients with type 2 diabetes for evidence of β-cell replication and sought to determine changes in expression of proteins involved in insulin signaling and β-cell cycle control using high resolution confocal imaging of pancreas sections and RT-PCR of islets isolated from diabetic patients. We have focused on proteins in the insulin/IGF-I signaling pathway and in cell cycle control since both pathways are critical for maintenance of adult cell mass. Furthermore, based on a mouse model showing similar defects, we provide mechanistic evidence for a role for insulin signaling and FoxO1 in the modulation of β-cell survival and proliferation.

## Materials and Methods

### Patients

The clinical characteristics and laboratory data of the group of patients with T2DM and controls are provided in Table 1 and Table 2. The pancreatic specimens were carefully selected in relation to their morphological quality and all routine immunohistochemical staining performed using antibodies directed against pancreatic hormones were subject to quality control using appropriate positive and negative controls. All specimens were fixed in buffered formalin (formaldheyde 4% and acetate buffer 0.05 M) for 24 hours and routinely embedded in paraffin. Five micrometer thick sections were stained with hematoxylin-eosin for morphological evaluation. Human pancreas specimens from donors were fixed and immunostained as previously described [8], [9], [35], [36].

**Table 1:** Non-diabetic multiorgan donors.

N°	AGE(YRS)	GENDER(M/F)	WEIGHT(Kg)	HEIGHT(m)	BMI(Kg/m2)	CAUSE OF DEATH
**1**	58	M	86	1,75	28,08	TRAUMA
**2**	72	F	70	1,70	24,22	CEREBRAL HEMORRHAGE
**3**	75	F	60	1,65	22,04	CEREBRAL HEMORRHAGE
**4**	67	F	65	1,65	23,88	CEREBRAL HEMORRHAGE
**5**	79	M	65	1,70	22,49	CEREBRAL HEMORRHAGE
**6**	73	M	80	1,80	24,69	CEREBRAL HEMORRHAGE
**7**	57	F	57	1,55	23,73	CEREBRAL HEMORRHAGE
**8**	63	M	55	1,65	20,20	CEREBRAL HEMORRHAGE
**9**	81	M	85	1,80	26,23	CEREBRAL HEMORRHAGE
**10**	55	M	75	1,80	23,15	CEREBRAL HEMORRHAGE
**11**	78	M	70	1,70	24,22	CEREBRAL HEMORRHAGE
**12**	82	M	70	1,70	24,22	CEREBRAL HEMORRHAGE
**13**	62	F	63	1,56	25,89	CEREBRAL HEMORRHAGE
**14**	73	M	70	1,70	24,22	CEREBRAL HEMORRHAGE
**15**	73	M	80	1,74	26,42	CEREBRAL HEMORRHAGE
**16**	69	F	65	1,55	27,06	CEREBRAL HEMORRHAGE
**17**	76	M	90	1,85	26,30	TRAUMA
**18**	68	M	85	1,8	26,23	CEREBRAL HEMORRHAGE
**MEAN**	**70,06**	**12M/6F**	**71,72**	**1,70**	**24,63**	
**± SEM**	**8,29**		**10,56**	**0,09**	**1,97**	

The details of the non-diabetic organ donors. Data are expressed as means ± SEM.

**Table 2:** Type 2 diabetic multiorgan donors.

N°	AGE (YRS)	GENDER (M/F)	WEIGHT (Kg)	HEIGHT (m)	BMI (Kg/m2)	CAUSE OF DEATH	KNOWN DURATION OF DIABETES	ANTIDIABETIC THERAPY
**1**	54	M	90	1,80	27,8	CEREBRAL HEMORRHAGE	N.A.	SULPHONYLUREA + METFORMIN
**2**	49	M	110	1,82	33,2	CEREBRAL HEMORRHAGE	N.A.	DIET
**3**	74	M	99	1,95	26,0	CEREBRAL HEMORRHAGE	8	METFORMIN
**4**	70	F	70	1,65	25,7	CEREBRAL HEMORRHAGE	5	SULPHONYLUREA + METFORMIN
**5**	66	M	75	1,80	23,1	CEREBRAL HEMORRHAGE	7	SULPHONYLUREA + METFORMIN
**6**	56	M	100	1,80	30,9	CEREBRAL HEMORRHAGE	3	DIET
**7**	70	M	90	1,79	28,1	CEREBRAL HEMORRHAGE	N.A.	SULPHONYLUREA
**8**	62	M	85	1,80	26,2	CEREBRAL HEMORRHAGE	N.A.	SULPHONYLUREA
**9**	67	F	70	1,75	22,9	CEREBRAL HEMORRHAGE	15	INSULIN
**10**	66	F	85	1,80	26,2	CEREBRAL HEMORRHAGE	20	INSULIN
**11**	75	F	80	1,65	29,4	TRAUMA	6	SULPHONYLUREA
**12**	75	F	75	1,65	27,5	ICTUS	11	SULPHONYLUREA + METFORMIN
**13**	63	M	90	1,75	29,4	CEREBRAL HEMORRHAGE	6	SULPHONYLUREA+METFORMIN+INSULIN
**14**	68	F	80	1,65	29,4	CEREBRAL HEMORRHAGE	N.A.	METFORMIN+INSULIN
**MEAN**	**65,36**	**8M/6F**	**85,64**	**1,76**	**27,56**		**9,00**	
**± SEM**	**7,92**		**11,82**	**0,09**	**2,83**		**5,43**	

The details of the diabetic organ donors. Data are expressed as means ± SEM.

### Ethical approval

All experiments in regard to use of human tissues were in compliance with and following approval of the Institutional Review Board of the Joslin Diabetes Center. Human pancreata were collected from brain-dead organ donors after informed consent was obtained in writing from family members, as previously reported [37]. The islet isolation centers had approval to isolate islets for scientific research if they were not considered suitable for clinical islet transplantation, and were in accordance with the Institutional ethical requirements. Animal experiments were performed in compliance with the Instituional Animal Care and Use Committee (IACUC) of the Joslin Diabetes Center, Brandeis University and Harvard Medical School and in compliance with the policies of the National Institutes of Health.

### Immunofluorescence by confocal and light microscopy

**Figure 1:**
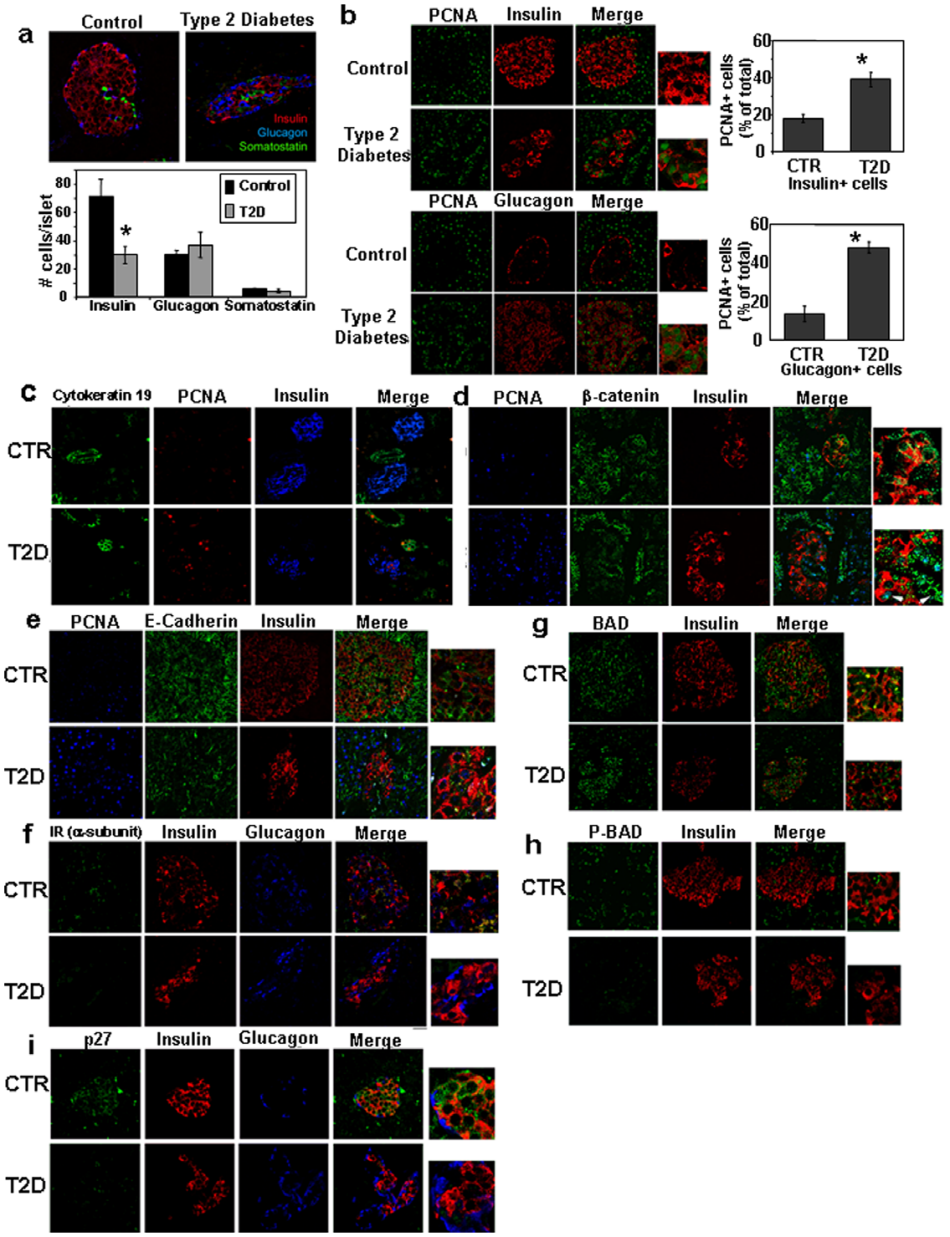
Reduced β-cell mass, enhanced PCNA+ β- and α-cells and altered expression of insulin signaling and cell adhesion proteins in pancreas from patients with T2DM. Representative confocal mages of pancreas sections from type 2 diabetic and control pancreas showing (a) quantification of islet cell number with immunostaining for insulin (red), glucagon (blue) and somatostatin (red); PCNA+ cells co-staining with (b) insulin or glucagons (red) and PCNA (green) and (c) CK19 (green), PCNA (red) and insulin (blue); (d) alterations in expression of β-catenin with immunstaining for PCNA (blue), β-catenin (green), insulin (red), light blue in the merged image indicates co-localization between β-catenin and PCNA; (e) alterations in E-cadherin with immunostaining for PCNA (blue), E-cadherin (green) and insulin (red) and the merged image in yellow; Immunostaining of pancreas sections for (f) insulin receptor α-subunit (green), insulin (red) and glucagon (blue); (g) BAD (green) and insulin (red) and merged image in yellow; (h) phospho-BAD (green), insulin (red) and merged image in yellow, and (i) the cell-cycle inhibitor p27-kip1 (green), insulin (red) and glucagon (blue), (*n* = 7). Yellow in merged images indicates co-localization between insulin staining and insulin signalling proteins. Immunostaining was performed as described in Methods. Bars = 50 µm. *, p<0.05 controls vs T2D.

Staining with primary antibody was followed by incubation with FITC-, TRITC, or CY5-conjugated secondary antibodies. Islets were imaged using a Bio-Rad MRC 1024 confocal laser scanning microscope. To reduce the bleed through from below, confocal images were acquired sequentially, using the LaserSharp2000 software with a low iris diameter. The florophores (FITC, TRITC and Cy5) are all commonly used for triple immunostaining and the bleed through for these fluorophores is negligible when sequential scanning is employed. Identical parameters (laser power, iris diameter and gain) were maintained to acquire images from all sections. Background signal due to non-specific binding was subtracted from “test” images. The confocal imaging techniques employed in these studies are consistent with previously published methods [8]. Immunofluorescence studies reported in figure 1c–i were performed on seven different control and T2D subjects and similar results were obtained. Islet numbers were evaluated by counting 10 fields at 40× magnification.

### Quantification of endocrine cells in healthy subjects and T2D patients

Analysis was performed on seven healthy controls and seven T2D subjects, and a total of 100 controls and 100 T2D islets were analysed. Two sections for each pancreas were triple stained with glucagon, insulin and somatostatin followed by incubation with FITC-, TRITC- and CY5-conjugated secondary antibodies. Fourteen to 16 islets per subject were randomly selected and imaged with a confocal microscope and the number of insulin-, glucagon- or somatostatin-positive cells per islet was manually counted in blind by two independent observers. Islets with less then 30 endocrine cells were not included in this analysis. The number of PCNA-positive cells was manually counted blind by two independent observers. Data are expressed as a percentage of total insulin-, glucagon- or somatostatin-positive cells per islet.

### Immunohistochemistry for Ki67

To evaluate Ki67 protein expression, immunohistochemical staining using the ABC peroxidase technique was performed on formalin fixed and paraffin embedded tissues [36]. Briefly, three micrometer thick sections were mounted on poly-L-lysine coated slides, de-paraffinized and hydrated through graded alcohol to water. Endogenous peroxidase activity was inhibited by dipping sections in 3% hydrogen peroxide for 10 minutes at room temperature. After heat antigen retrieval in a microwave oven, primary Ki67 antibody (monoclonal, clone MIB1 at 1∶100, Dako, Copenhagen) incubation was performed at 4°C for 18–20 hours and was followed by the avidin-biotin complex (ABC) procedure. Sections were then immersed in 0.03% 3,3′ diaminobenzidine tetrahydrochloride and counterstained with Harris' hematoxylin.

### RT-PCR

Isolated islets were purified from five type 2 diabetic subjects and seven normoglycemic controls using the modified Ricordi method as described in Gunton *et al*. Semi-quantitative-PCR of insulin receptor isoforms was performed as described previously [19].

### Western blotting of islets from patients with T2DM

Insulin receptor (IR), insulin receptor substrate-1 (IRS1), insulin receptor substrate 2 (IRS2) and phosphatidylinositol 3-kinase p110 alpha (PI3K) expression was determined by immunoblotting as reported previously [38]. Aliquots of islet cell lysates obtained from 400 hand-picked islets and containing 250 µg protein, were immunoprecipitated by incubation with antibodies against total (anti-IR antibody, provided by G. Sesti; anti-IRS1 antibody, Santa Cruz Biotechnologies, CA, USA; sc-17200; anti-IRS2 antibody, Millipore, Massachusetts, USA; 06-506; and anti-PI3K, Santa Cruz Biotechnologies, CA, USA; sc-7174) and activated (anti IRS-1/2, Tyr 612, Santa Cruz Biotechnologies, CA, USA; sc-17195) proteins. After immunoprecipitation, bound antibodies were detected using procedures performed according to the manufacturer's instructions (ECL, Amersham Biosciences, Buckinghamshire, UK). Bands of interest were quantified by a densitometer (GS 690 BioRad, Laboratories CA, USA) using a MultiAnalyst/PC-PC Software for Bio-Rad's Image Analysis Systems, Version 1.02 (BioRad Laboratories CA, USA).

### Animals, cell lines and DNA content analyses

β-cell specific insulin receptor knockout (βIRKO) mice and control mice (mice expressing *Cre* recombinase on the rat insulin promoter or RIP-*Cre*) were generated as reported earlier [39]. Cell lines from Control and βIRKO mice were derived as described previously [40] . For islet studies, islets were isolated by the intraductal technique [40]. Islets and cultured β-cells from control and βIRKO mice were synchronized with hydroxyurea. Briefly, freshly isolated islets were cultured for 48 hours in RPMI-1640 medium containing 7 mM glucose and 10% FBS. The islets were then exposed to culture media containing 10 mM hydroxyurea for 16 hours. The hydroxyurea was removed by carefully rinsing islets in RPMI 1640 media before continuing culture. For DNA content, cells were collected by trypsinization, washed with PBS, fixed with 70% ethanol and kept at −20°C until analysis. The fixed cells were washed with PBS, stained with propidium iodide solution and analyzed using flow cytometry. Cell-cycle profiles were determined by Cell Quest and ModFit softwares. Insulin receptors were re-expressed in βIRKO cells as reported earlier [41].

### Cytosolic and nuclear fractions and immunoblotting

Cytosolic and nuclear fractions were prepared by lysing cells [42]. The fractionated lysates were subjected to immunoblotting and visualized by an enhanced chemiluminescence system (Roche). Antibodies recognizing Akt, FoxO1, p-FoxO1, cell-cycle proteins and PTEN were purchased from Cell Signaling (Beverly) and p27-Kip1 from BD Biosciences (San Diego). Antibodies to Cyclin D2, Cyclin D3 and Cyclin E were obtained from Santa Cruz, and to IRS-1, IRS-2 and phosphotyrosine (4G10) were from Upstate (Waltham).

### Statistical Analyses

All data are expressed as means + SEM. Differences between control and diabetic groups were determined using Student's independent ‘t’ test or ANOVA as appropriate and ‘*P’*<0.05 was considered significant.

## Results

To evaluate the relative proportion of islet cells we immunostained pancreas sections with antibodies against insulin, glucagon and somatostatin. We did not observe differences in islet numbers between groups. Consistent with earlier reports, we observed a 56% decrease in β-cells mass (p = 0.03) and an ∼8% increase in α-cell mass that did not reach statistical significance (p = 0.41) while somatostatin cell mass was similar (p = 0.68) in pancreas sections from patients with T2DM compared to controls (Fig. 1a) [14], [15], [16].

A large number of PCNA+ (proliferating nuclear cell antigen) cells that co-stained with insulin or glucagon in sections from diabetic patients indicated that both β- and α-cells are engaged in cell-cycle progression (Fig. 1b). In addition, in T2DM pancreases PCNA+ cells that co-stained for cytokeratin 19, a marker of exocrine cells, indicated proliferation in duct cells (Fig. 1c). The lack of significant alterations in PCNA+ cells that co-stained with somatostatin suggested the effects are specific to α- and β-cells (data not shown). In addition, we did not detect Ki67-immunoreactivity in islets cells in diabetic pancreases (data not shown).

β-cell regeneration in rodents has been reported to involve a process of de-differentiation and redifferentiation or epithelial-to-mesenchymal-like transition (EMT) [28], [30]. Whether this occurs *in vivo* in humans is unknown. We therefore immunostained pancreas sections with antibodies against E-cadherin and β-catenin – two important markers of the cadherin-catenin adhesion family of proteins known to be involved in EMT [43]. E-cadherin was clearly detectable in membranes of β-cells in the controls but reduced in the diabetic group (Fig. 1d). By contrast, β-catenin was co-localized with PCNA in the nucleus only in the diabetic group (Fig. 1e).

**Figure 2:**
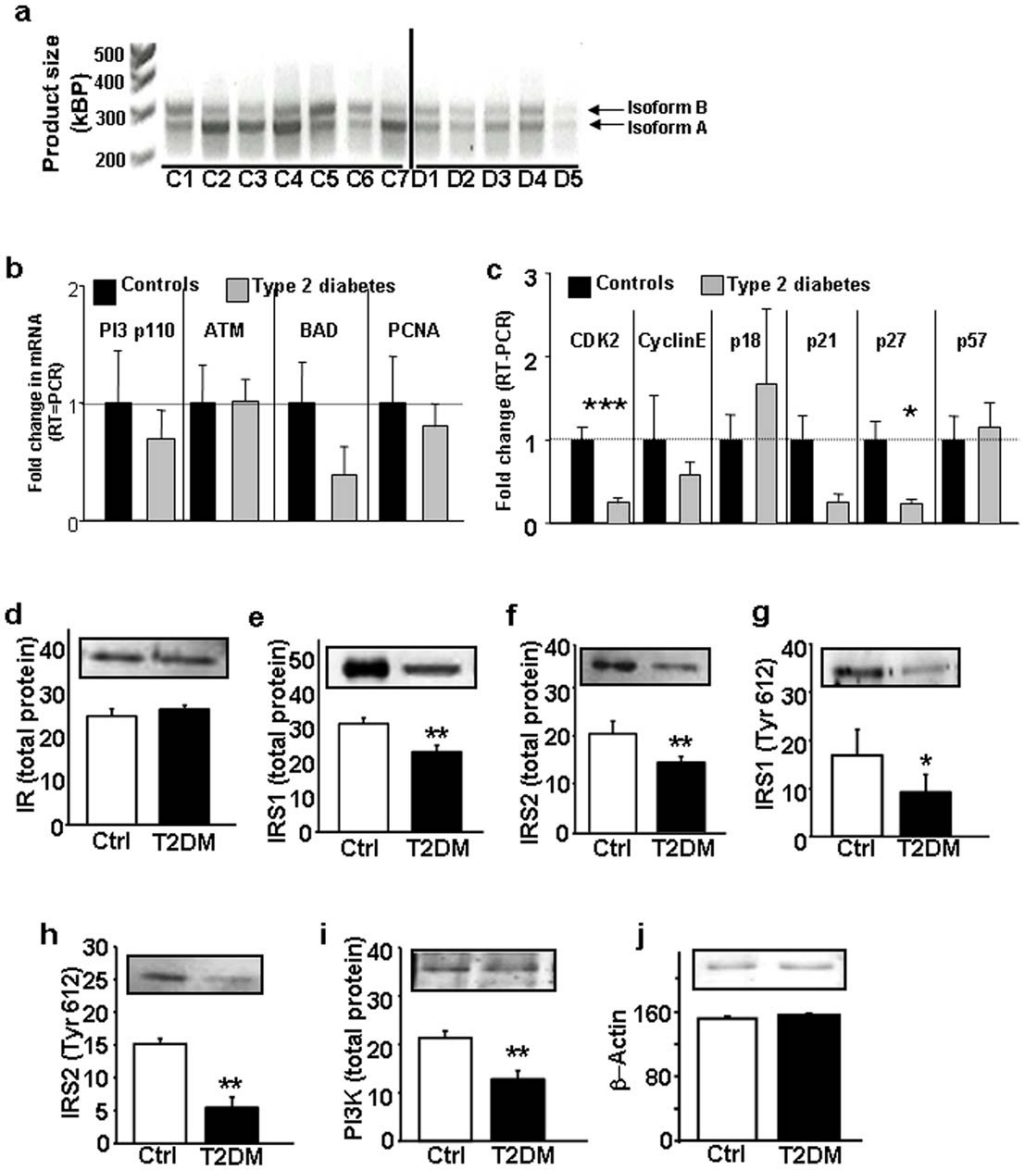
Reduced transcript levels of insulin signaling and cell-cycle proteins and protein levels of insulin signaling components in islets isolated from patients with T2DM. (a) Expression of insulin receptor isoforms A and B; (b) transcript levels of insulin signaling proteins, and (c) transcript levels of cell-cycle proteins. Data are expressed after normalization for TATA binding protein (TBP) (*n* = 5–7); *, p<0.05; ***, p<0.001; controls vs T2DM. (d) protein expression of total insulin receptors, (e), total insulin receptor substrate-1, (f) total insulin receptor substrate-2, (g), tyrosine-612 IRS-1, (h) tyrosine-612 IRS-2, (i) or total phosphatidyl inositol 3-kinase. *,p<0.05; **,p<0.001 Control vs T2D. Data in 2d–i are immunoprecipitates. (j) β-actin was used to normalize the data in Figures 2d–i.

Insulin and IGF-I signaling pathways play important roles in modulating the function/proliferation of islet β-cells and disruption of insulin receptors in mouse β-cells leads to a phenotype that mimics human T2DM [39], [44]. A reduced expression in insulin receptor levels was evident in islets and acinar tissues of patients with T2DM (Fig. 1f). Next, we examined expression of downstream signaling proteins BAD and phospho-BAD that are known to be important in the apoptotic pathway [45]. While BAD protein was expressed in both groups, p-BAD protein was virtually absent in β-cells in the diabetic group suggesting enhanced apoptosis in the pancreases from the type 2 diabetes cases who also exhibited blunted insulin signaling (Fig. 1g,h). Considering the significant increase in PCNA+ β-cells in patients with diabetes, we then examined alterations in expression of proteins that regulate cell-cycle. Consistent with our findings of reduced transcript levels for the cell-cycle inhibitor, p27-kip1, we detected a marked decrease in immunostaining for the protein in diabetic β-cells (Fig. 1i). To examine the alterations in insulin receptor expression, we extracted RNA from islets isolated from patients with T2DM and controls, and subjected them to RT-PCR. Insulin receptors exist as two isoforms in mammalian tissues – A and B [9], [46]. Consistent with our earlier reports, we observed the expression of both A and B isoforms were significantly reduced in diabetes patients compared to controls (Fig. 2a) [19]. However, when the data were expressed as a ratio of the two isoforms we did not observe significant differences between groups (normal 0.63±0.1 vs diabetes 0.56±0.03, p = NS). Examination of cell-cycle proteins showed a decrease in expression of the cyclin dependent kinase cdk2, p27-kip1 and a trend towards a decrease in expression of p21 (p = 0.08) in the diabetic group (Fig. 2b,c). We examined the alterations in the expression of proteins in the insulin signaling pathway in islets isolated from controls and patients with T2D. We did not observe significant alterations between groups in the expression of insulin receptors suggesting a differential regulation of the mRNA and protein (Fig. 2d). However, the expression of both total and tyrosine-612 levels of IRS-1 and IRS-2 (Fig. 2 e–h) and total PI3-kinase (Fig. 2i) were significantly lower in T2D cases.

These data demonstrate a significant alteration in expression of insulin signaling proteins multiple cell-cycle proteins that potentially contributed to an inadequate β-cell hyperplastic response to overcome insulin resistance, and/or enhanced the susceptibility of β-cells to apoptosis, in the patients with diabetes.

### Insulin signaling in β-cell cycle regulation

To gain insight into the molecular pathways that link β-cell-cycle with insulin signaling, we took advantage of a mouse model lacking functional insulin receptors in β-cells (βIRKO). A notable feature of the βIRKO mouse is a decrease in β-cell mass suggesting that absence of insulin signaling directly influences β-cell regeneration in the mutants [39], [44]. Therefore we studied β-cell lines and isolated islets derived from three control and three knockout mice using previously described methods [40].

**Figure 3:**
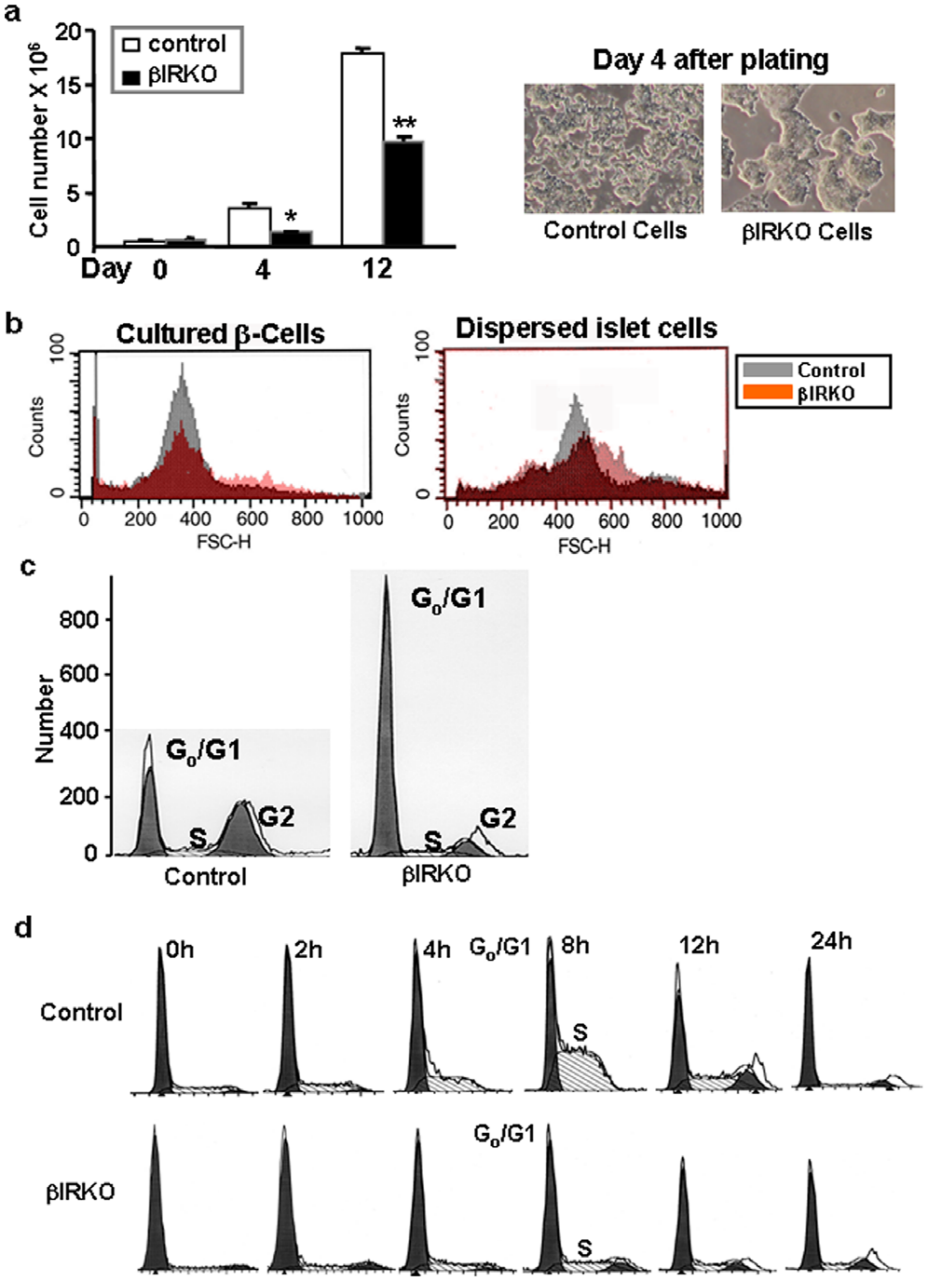
Insulin receptor-deficient β-cells exhibit slow growth and block in G1 to S-phase transition. (a) Defects in growth of βIRKO cells is evident from total number of cells 4 and 12 days after the same number of cells are plated at day 0; the panel on the right shows poor spread and clumping of βIRKO cells (b) β-cell size in control and βIRKO cell lines and islets determined by FACS analyses with forward scatter; (c) significantly lower G1 and G2 in βIRKO cells, and (d) block in G1 to S-phase transition in βIRKO cells after synchronization with hydroxyurea for 18h. Analyses were performed by FACS. In each case, representative data from 3 clones each derived from 3 RIP-*Cre* controls and 3 βIRKO mice are shown.

A striking feature of β-cells derived from βIRKO mice was the extremely slow growth compared to control β-cell lines (Fig. 3a). The absence of significant differences in size prompted us to examine cell-cycle control in the βIRKO group (Fig. 3b). Indeed, DNA content analysis using propidium iodide staining of βIRKO cells revealed a higher Go/G1 phase and reduced G2 population, suggesting an impaired ability to transit from G1 to S phase (Fig. 3c). To evaluate this possibility we synchronized cultured β-cells by hydroxyurea treatment for 16 hours and released the cells from quiescence by culturing cells in the presence of serum. Eight hours after release, ∼62% of control cells entered S phase compared to only ∼18% of βIRKOs, providing direct evidence that the slow growth of mutant β-cells is due to G1 to S phase arrest (Fig. 3d).

**Figure 4:**
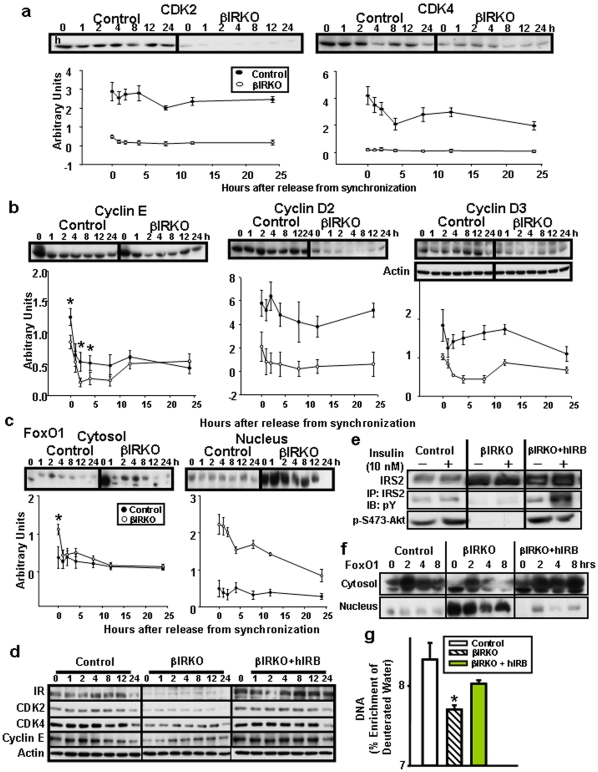
Insulin receptor-deficient β-cells show reduced expression of cyclins and cyclin dependent kinases and nuclear restriction of FoxO1. (a) Reduced expression of cdk2 and cdk4; (b) reduced expression of cyclin D2, cyclin D3 and cyclin E; (c) increased expression and nuclear restriction of FoxO1; (d) re-expression of the insulin receptor in βIRKO β-cells (βIRKO+hIRB) restores the expression of CDK2, CDK4 and cyclin E. Actin is used as a loading control; (e) reduced p-IRS2 and p-S473-Akt in βIRKO and restoration of total IRS2, pIRS2 and p-S473-Akt in insulin receptor re-expressing cells (βIRKO+hIRB); (f) Re-expression of insulin receptors in βIRKO cells restores FoxO1 to nucleus; (g) proliferation analyzed by the deuterated water technique [57]. Representative data from at least 3 independent Western blotting experiments from at least 2 independent clones are shown. The plots below the blots are shown in arbitrary units. The loading control for experiments in Fig. 4a–c is presented under the blot for cyclin D3 in Fig. 4b. *, p<0.05 βIRKO vs Control or βIRKO+hIRB.

To analyze the expression of proteins in regulation of cell-cycle progression we examined cyclins, cyclin-dependent kinases (CDK) and CDK inhibitors [47]. Similar to findings in the diabetic group, the levels of expression of CDK2 were virtually absent in cultured βIRKO cells, and CDK4, an important regulator of cell-cycle was also significantly down-regulated (∼85%) in mutant β-cells (Fig. 4a). We also observed a >80% reduced expression in cyclin D2 and D3 and a 42% reduction in cyclin E expression (Fig. 4b) [25]. Consistent with data in human pancreas, we observed a slight but significant increase in PCNA+ β-cells in βIRKO islets (0.11±0.04 vs 0.17±0.03% β-cells, *n* = 3, p<0.05) suggesting an attempt by the cells to proliferate. Together, these changes are consistent with a block in cell-cycle progression in βIRKO β-cells.

To identify key proteins that link insulin signaling with β-cell proliferation we explored the expression and nuclear localization of FoxO1. Protein levels for FoxO1 were up-regulated by three to five-fold and restricted to nuclear fractions in βIRKO cells (Fig. 4c). The fraction of FoxO1 that was phosphorylated was also significantly lower in β-cells of the βIRKO mice in cytosolic (14±8 vs 81±18%; *n* = 3, *p*<0.05) and nuclear fractions (18±11 vs 68±16%; *n* = 3, *p*<0.05). Thus, a lack of functional insulin receptors in β-cells leads to significantly reduced phosphorylation and nuclear restriction of FoxO1.

To directly rescue the defects observed in the βIRKO cells, we established stable β-cell lines expressing the human insulin receptor B isoform (βIRKO-hIRB cells) [40], [48]. Expression of CDK2, CDK4 and cyclin E proteins (Fig. 4d) and tyrosine phosphorylation of IRS2 and p-S473-Akt were restored (Fig. 4e) and expression of FoxO1 was significantly reduced in nucleus and increased in cytosolic fractions (Fig. 4f). Consequently, proliferation of insulin receptor re-expressing β-cells showed a significant increase in DNA synthesis compared to βIRKO cells (Fig. 4g).

## Discussion

Consistent with earlier reports [14], [15], [16], [17], we observed reduced β-cells and increased α-cells in patients with type 2 diabetes. Interestingly, we detected a striking increase in the number of α- and β-cells that co-stained with the proliferation marker, PCNA, suggesting that both α- and β -cells are attempting to enter the cell cycle. These data may have important implications for therapeutic efforts to counter hyperglycemia in patients with type 1 or type 2 diabetes that are focused on the regenerative potential of β-cells.

Insulin/IGF-I signaling mediates diverse pathways to modulate proliferation and anti-apoptosis in most mammalian cells including pancreatic islets [3], [13] . Recent studies in humans and in rodent models of insulin resistance, diabetes and obesity implicate an important role for insulin/IGF-I signaling in β-cell biology [9], [13], [33], [39], [40], [41], [44], [46]. The significant decrease in insulin receptor expression in islets from patients with type 2 diabetes indicates that blunted insulin signaling in β-cells may make them susceptible to apoptosis [9], [19]. Alterations in phosphorylation of the pro-apoptotic protein BAD are known to modulate apoptosis. Thus, a reduced phosphorylation of BAD in the diabetic pancreas is consistent with an increased apoptosis in type 2 diabetes that possibly occurs, in part, secondary to hyperglycemia [8], [15]. In contrast, the higher α-cell mass may reflect the resistance of α-cells to apoptosis in patients with type 2 diabetes and is associated with higher circulating levels of glucagon [6], [7], [9], [17]. We have previously reported that human islets that have been transplanted in streptozotocin-treated diabetic SCID mice exhibit α-cells that are resistant to hyperglycemia-induced apoptosis, in contrast to the apoptosis-susceptible β-cells [49], [50].

Studies in rodents indicate that β-cell replication is a major mechanism that contributes to maintaining adult β-cell mass [22], [25], [28] Our observations of a striking increase in the number of PCNA+ cells clearly indicates that human islet cells are also capable of entering the cell cycle. The significant increase in the number of PCNA+ β-cells in the diabetic group indicates that either the β-cells are attempting to replicate as a compensatory response to peripheral insulin resistance and/or that the increase in PCNA expression is a DNA repair response to overcome the effects of pro-apoptotic stimuli including elevated circulating levels of glucose and free fatty acids - a consistent pathological feature of type 2 diabetes [51], [52], [53]. It is worth noting that replicating β-cells are more susceptible to cell death induced by islet amyloid polypeptide that accumulates in β-cells in patients with type 2 diabetes [52]. Furthermore, consistent with the findings regarding an increase in PCNA expression in conditions of cell stress, in Affymetrix gene expression studies, we have observed a 40-fold increase in the expression of PCNA mRNA in human islets of Langerhans that have been cultured for five days as compared to 24 hours (Folli F, Perego L, Davalli A, unpublished observations), a condition in which significant β-cell apoptosis can be detected [8], [49], [50]. The lack of significant differences in PCNA mRNA in islet samples (Fig. 2a) may be due to presence of multiple cell types in islets and/or differential regulation of PCNA at the transcriptional versus post-translational levels in β-cells [54]. While these possibilities are not mutually exclusive, the reduced β-cell mass clearly indicates an abortive attempt of the PCNA+ cells to progress through the cell cycle and develop into functional β-cells with a normal life span. The down regulation of key cell cycle proteins including p27-kip1 and cdk2 in the diabetic pancreas provides additional evidence for altered islet cell cycle dynamics that could promote a default pathway towards apoptosis in β-cells. For example, p27-kip1, in addition to inhibiting cyclins also acts as an anti-apoptotic factor and the low expression of the protein in β-cells may accelerate the apoptotic process [55], [56]. This is compounded by a concomitant reduction in the expression of CDK2, CDK4 and cyclin E proteins, which are essential for multiple steps in the transition from G1 to S phase of the cell cycle.

In addition to anti-apoptosis, insulin signaling regulates the transcription factor FoxO1 that, in turn, interacts with PDX-1 to modulate β-cell proliferation [29]. The near complete reversal of nuclear restriction of FoxO1 and rescue of blunted proliferation by re-expression of the insulin receptor in βIRKO cells [57] indicates a direct link between insulin signaling and β-cell-cycle control. FoxO proteins, including FoxO1, have been implicated in cell cycle regulation [58]. For example, stress-induced FoxO activation has been reported to alter the expression of genes that contribute to cell cycle arrest [59]. Additional studies are necessary to investigate the proteins that are directly activated by FoxO1 to modulate islet cell cycle progression. Free fatty acids are also known to modulate expression of insulin signaling proteins *in vitro* [53]. Although we did not observe alterations in expression of enzymes involved in lipid metabolism in diabetic islets (data not shown), it is possible that ectopic lipid deposition in islets could produce some of the changes observed in the diabetic group and requires further study.

**Figure 5:**
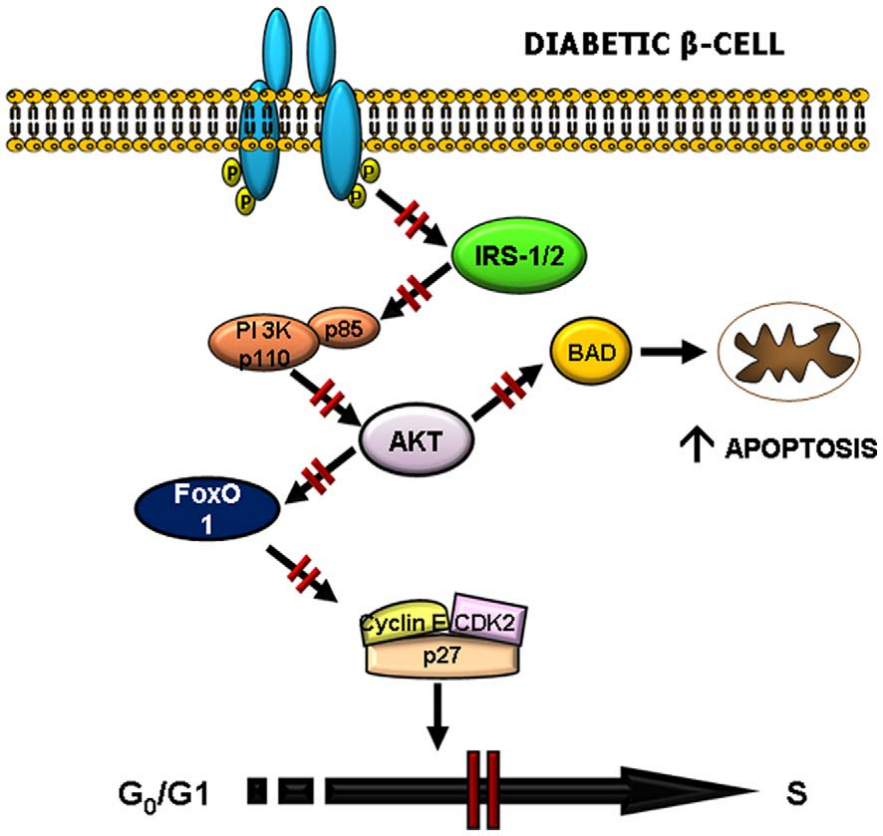
Schematic linking the growth factor pathway to defects in cell cycle progression and apoptosis in diabetic β-cells.

In conclusion, we propose that β-cells in patients with T2DM are able to enter the cell-cycle, but fail to proliferate successfully to compensate for peripheral insulin resistance due to dysfunctional insulin signaling and cell-cycle arrest (Fig. 5). Restoration of insulin signaling and cell-cycle control in β-cells may be one approach to plan therapeutic strategies to counter β-cell loss in T2DM.
